# A scoping review of FGM in humanitarian settings: an overlooked phenomenon with lifelong consequences

**DOI:** 10.1186/s13031-022-00479-5

**Published:** 2022-09-15

**Authors:** Shatha Elnakib, Janna Metzler

**Affiliations:** 1 Johns Hopkins Bloomberg School of Public Health, Baltimore, MD USA; 2 Women’s Refugee Commission, New York, USA

**Keywords:** Female genital mutilation, Sexual and reproductive health, Humanitarian, Conflict, Crises, Emergencies, Scoping review

## Abstract

**Background:**

Female genital mutilation (FGM) is widely recognized as a human rights violation. Little is known about FGM rates and practices in humanitarian settings, and about the impact of crisis on the drivers and consequences of FGM. This scoping review set out to investigate the current research landscape on FGM in humanitarian settings.

**Methods:**

We conducted a search of electronic databases and gray literature published between 1990 and 2021. This was coupled with backward citation tracking on eligible studies and reviews. We analyzed studies that met our eligibility criteria using thematic analysis.

**Results:**

We found 13 peer-reviewed and four grey literature articles. Most studies were published in the last decade between 2010 and 2021, signaling growing attention to the issue. Five of the 17 articles provided estimates of incidence based on primary data collection amongst crisis-affected populations, ten focused on drivers, ten on consequences and five on interventions. The limited studies that have examined FGM in humanitarian settings indicate that the impact of crisis on FGM is multifaceted and context-specific, depending in part on interactions with host and other displaced communities and their social norms and practices. There is evidence that the acquisition and transfer of harmful social norms may take place during migration flows, but also that social norms underlying FGM may weaken in contexts of displacement, causing the practice to decrease. The incidence of FGM may also remain unchanged, but the type of FGM practiced may shift from more harmfully perceived forms to less radical forms. We found that drivers of FGM may be exacerbated, attenuated, or unchanged by crisis and displacement. Overall, there was predominant focus on medical consequences of FGM, and limited research on the social, economic, and psychological consequences of the practice. There was also a dearth of research into intervention effectiveness.

**Conclusions:**

Despite an increase in research on FGM in humanitarian settings, there is still a notable dearth of studies investigating the impact of emergencies on FGM and the factors that propel it. More research and documentation of evidence are needed to inform interventions and policies.

**Supplementary Information:**

The online version contains supplementary material available at 10.1186/s13031-022-00479-5.

## Background

Female genital mutilation (FGM), defined as the partial or total removal or injury of the female external genitalia for reasons that are non-medical, is widely recognized as a human rights violation [1]. Its elimination is included as a target in the Sustainable Development Goals, as part of goal 5 to achieve gender equality and empower women and girls [2]. Globally, around 200 million girls have been subjected to the practice, and according to UNICEF, more than 4 million girls are at risk of undergoing the procedure annually [3]. The COVID-19 pandemic is causing disruptions in FGM programmes that are projected to result in an excess 2 million FGM cases in the next ten years that would have otherwise not occurred if it weren’t for the pandemic [4]. Thus, there is renewed urgency to accelerate progress towards curbing the practice.

FGM has detrimental consequences for girls’ and women’s physical and mental health and social wellbeing. It can cause hemorrhage, urinary problems, as well as infections [5]. In many cases, the procedure can result in death. Moreover, during childbirth, the procedure can cause complications and newborn death. There is more limited evidence on the impact of FGM on social and economic outcomes [6, 7]. However, available evidence indicates that FGM has some impacts on school attendance and productivity, with links between FGM and school dropout due to its association with marriage which serves as an alternative to schooling. In addition, because FGM may cause health complications, it may increase school absenteeism and drop-out [5, 7, 8]. Adverse consequences of FGM on women’s psychological wellbeing include associations with post-traumatic stress disorder which have been found among women in their home countries as well as among immigrants in the west, and other forms of psychological trauma [9].

In many settings, the harmful practice is considered a rite of passage to womanhood, and is mostly practiced among girls under age 15 [10]. It is perpetuated by deeply entrenched gender and social norms which also work to curtail women’s power in society, reduce their access to educational opportunities, and limit their participation in the labor force [3].

The impacts of humanitarian emergencies are inherently gendered, disproportionately impacting women and girls. Gender inequalities and gender-based violence tend to be exacerbated in these situations due to disruptions in social and community networks, breakdown of families, and closures of schools and health facilities [11]. While 35% of women worldwide report experiencing gender-based violence, more than 70% of women experience gender-based violence in some humanitarian contexts [12].

While it can be expected that humanitarian emergencies increase the incidence of FGM, limited research has empirically investigated the impact of emergencies on FGM rates. Moreover, little is known about how the drivers of FGM are altered, exacerbated, or attenuated in these contexts, nor about the unique consequences of FGM created by emergency situations and the opportunities to prevent FGM among crisis-affected and displaced populations.

Against this backdrop, this scoping review was conceived. The overall research question guiding our review is “What is the evidence base on FGM in humanitarian settings?” Specific research questions include the following: (1) what is the evidence available on the prevalence of FGM in humanitarian settings, including on shifts in prevalence stemming from conflicts, natural disasters, or situations of forced displacement; (2) how do humanitarian emergencies modify the pre-existing drivers of FGM, if at all; (3) what are the unique complications arising from FGM in humanitarian settings; and (4) what are promising interventions—both policy-oriented and programmatic—that aim to prevent or respond to FGM in humanitarian settings.

## Methods

Our review was guided by the scoping review methodological framework developed by Arksey and O’malley [13]. Scoping reviews are studies that ‘aim to map rapidly the key concepts underpinning a research area and the main sources and types of evidence available’ [13, 14]. Because the purpose of scoping reviews is to characterize the breadth of the existing literature on a given topic, they lend themselves to broad research questions [15]. Given our interest in analyzing the current evidence base on FGM in humanitarian settings, we selected this methodology to guide our analysis.

We followed the five stages proposed by this framework:

1. Stage 1: identifying the research question;
2. Stage 2: identifying relevant studies;
3. Stage 3: study selection;
4. Stage 4: charting the data;
5. Stage 5: collating, summarizing and reporting the results.

This scoping review follows the reporting requirements set out by the Preferred Reporting Items for Systematic reviews and Meta-Analyses extension for Scoping Reviews (PRISMA-ScR) Checklist [16]. To ensure methodological rigor and replicability, our scoping review followed a pre-specified research protocol that described the review aims and methods for both the published and grey literature. The protocol is available upon request.

### Search strategy

A comprehensive literature search was conducted to identify all relevant studies addressing FGM in humanitarian settings. We developed a broad search strategy to maintain breadth of coverage. Using a combination of keywords, we searched a total of four electronic databases: Pubmed, Embase, PsycINFO, and Web of Science for articles published from January 1, 1990 to August 31, 2021. This was coupled with backward citation tracking on eligible studies and reviews. The search strategy was reviewed by a medical librarian at Johns Hopkins University and search terms were adapted for each database (Additional file 1).

To identify grey literature, we conducted a targeted search of publications posted on the websites of select international organizations working on FGM in humanitarian settings. Additionally, we searched the reference lists of selected articles to identify additional publications that may have been missed.

### Inclusion/exclusion criteria

Articles were included if they met the following criteria:

- Studies focusing on FGM prevalence, drivers, consequences, and interventions
- Studies conducted in the context of a humanitarian emergency (the acute, chronic, or early recovery phases of a crisis) or among a displaced population in a LMIC
- Studies conducted in English, Arabic and French
- Studies published from 1990 onward given that the issue of sexual and reproductive health and rights in humanitarian settings gained prominence in the 90’s.

The following exclusion criteria were used:

- Non-English, non-French, and non-Arabic articles.
- Books, poster presentations, dissertations, and articles without abstracts
- Studies conducted in high-income settings

### Article selection

SEN imported articles into *Covidence* software. SEN and JM screened titles and abstracts of all publications identified in the first stage of the review, and chose candidate articles for full-text review. SEN and JM independently reviewed the retrieved full-text articles to ascertain their eligibility for inclusion in the review. Where conflicts or disagreements arose in the study selection, the two reviewers discussed the conflicts until consensus was reached and the discrepancy was resolved.

### Charting the data

Data were extracted for all included studies using a charting form sheet. Data entry was done in Excel and the extraction included study title, year of publication, setting and geographic region, target population, sample size, research methodology, as well as information about the publication type, and the main findings of the study.

### Data analysis

We organized our analysis using a pre-specified framework that classifies FGM research outputs into four key areas: (1) prevalence or incidence of FGM; (2) factors associated with FGM; (3) consequences of FGM; (4) interventions aiming to prevent FGM or to support survivors. We used Excel to generate descriptive statistics, and thematic analysis guided our narrative write-up.

## Results

**Fig. 1:**
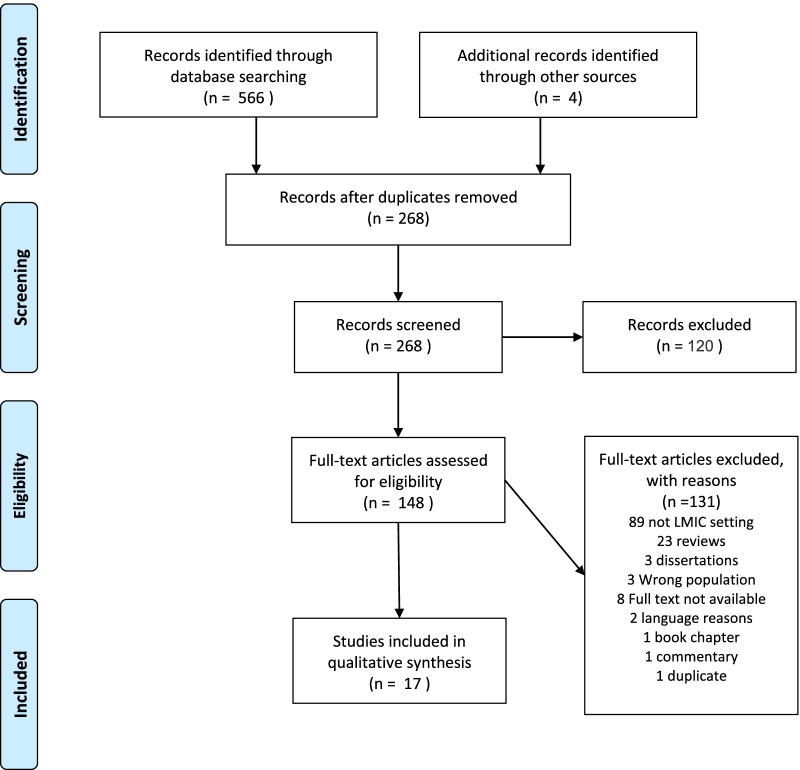
PRISMA diagram

A total of 507 studies were retrieved and imported into Covidence software. After the removal of duplicates, 265 articles were screened for title and abstract, and 113 full-text studies were assessed for eligibility. There were seventeen reviews identified. The reference list of each of the 17 reviews was checked and eligible articles were retrieved and imported into Covidence software. Of the 113 articles, 17 articles were eligible for inclusion. The remaining articles were excluded owing to the reasons listed in Fig. 1. Most studies were excluded because they examined FGM among refugees and asylum seekers in high-income countries, which was outside the scope of this review.

Of the 17 articles included in the review, 4 were quantitative, 5 were qualitative, and 2 were mixed methods studies featuring primary data collection as a means of responding to evidence gaps, 6 were policy briefs or technical reports providing recommendations for interventions based on desk reviews, case studies, or anecdotal reports (Table 1).The majority of studies (12 out of 17) were published in the last decade between 2010 and 2021, two studies were published between 1990 and 2000, and the remaining three studies were published in the 2000’s. Four studies were conducted in Kenya (one of which was a multisite study conducted with Somali women in both South Africa and Kenya), 3 in Sudan, 1 in South Africa, 1 in Ethiopia, 1 in Iraq, 1 in Cote D’Ivoire, and 1 in Uganda. The remaining publications presented evidence from case studies conducted in several countries.

**Table 1:** Characteristics of studies investigating FGM in humanitarian settings

References	Title	Year	Country	Setting	Study type	Study focus
Campbell and Sham [28]	Sudan: situational analysis of maternal health in Bara District, North Kordofan	1995	Sudan	Bara, Gerejikh and Taiyba rural councils	Qualitative	Consequences
Cohen [30]	The Reproductive Health Needs of Refugees: Emerging Consensus Attracts Predictable Controversy	1998	NA	NA	Policy brief	Intervention (the release and field testing of the Inter-Agency Field Manual for Reproductive Health in Refugee Situations)
Gately [27]	Sudan: A Humanitarian Response to a Silent Genocide: An American Nurse’s Perspective	2005	Sudan	Darfur	Qualitative	Drivers and consequences
Furuta and Mori [23]	Factors affecting women's health-related behaviors and safe motherhood: a qualitative study from a refugee camp in eastern Sudan	2008	Sudan	Um Gargur (refugee camp), Gedaref, Eastern Sudan	Qualitative	Drivers
Mitike and Deressa [18]	Prevalence and associated factors of female genital mutilation among Somali refugees in eastern Ethiopia: a cross-sectional study	2009	Ethiopia	Aysha, Kebribeyah, and Hartishek refugee camps	Quantitative	Prevalence and drivers
Khalife [24]	"They took me and told me nothing": female genital mutilation in Iraqi Kurdistan	2010	Iraq	Iraqi Kurdistan	Qualitative	Drivers and consequences
Plo et al. [17]	Female Genital Mutilation in Infants and Young Girls: Report of Sixty Cases Observed at the General Hospital of Abobo (Abidjan, Cote D’Ivoire, West Africa)	2014	Cote D'Ivoire	Abidjan	Quantitative	Prevalence
Ryan et al. [20]	The impact of emergency situations on female genital mutilation	2014	Multi-site case studies	Multi-site case studies	Evidence Synthesis	Prevalence, drivers, consequences, and interventions
Jinnah and Lowe [26]	Circumcising circumcision: renegotiating beliefs and practices among Somali women in Johannesburg and Nairobi	2015	South Africa, Kenya	Urban-Johannesburg and Nairobi	Qualitative	Drivers
Nyoka et al. [36]	Sanitation practices and perceptions in Kakuma refugee camp, Kenya: Comparing the status quo with a novel service-based approach	2017	Kenya	Kakuma refugee camp	Mixed Methods	Interventions
Im et al. [29]	Polyvictimization and mental health consequences of female genital mutilation/circumcision (FGM/C) among Somali refugees in Kenya	2019	Kenya	Eastleigh (Little Mogadishu), urban business district	Quantitative	Consequences
Ivanova et al. [19]	A cross-sectional mixed-methods study of sexual and reproductive health knowledge, experiences and access to services among refugee adolescent girls in the Nakivalerefugee settlement, Uganda	2019	Uganda	Nakivale refugee settlement	Mixed Methods	Prevalence, drivers, and consequences
Murewanhema [35]	Adolescent girls, a forgotten population in resource-limited settings in the COVID-19 pandemic: implications for sexual and reproductive health outcomes	2020	NA	NA	Policy Paper	Drivers and consequences
UNICEF [21]	The Humanitarian-Development Nexus: The future of protection in the elimination of female genital mutilation	2020	Multi-site case studies	22 countries under joint programe	Evidence Synthesis	Prevalence and interventions
The Community of Practice on FGM [22]	Preventing and responding to female genital mutilation in emergency and humanitarian contexts results from the virtual international stakeholder dialogue	2020	Multi-site case studies	NA	Evidence Synthesis	Prevalence, drivers, and consequences
Wenzel et al. [34]	FGM and restorative justice—a challenge for developing countries and for refugee women	2021	NA	NA	Concept paper with evidence synthesis	Drivers, consequences and interventions
Swan and Im [25]	Predicting mental health outcomes in a sample of Somali refugee youth: The role of child trauma	2021	Kenya	Eastleigh (Little Mogadishu), urban business district	Quantitative	Consequences

### Prevalence or incidence of FGM and shifts during displacement

The incidence of FGM in humanitarian settings and how the practice is impacted by conflict, forced displacement and climate-related disasters remains largely unknown. Three of the 17 articles provided prevalence estimates based on primary data collection amongst crisis-affected populations. Other estimates of prevalence noted throughout the remaining articles were relegated to DHS, MICS or National Statistics data, and largely unrepresentative of rates incurred amongst populations forcibly displaced due to conflict or natural disaster.

In a hospital-based sample (n = 409) in Abidjan, Cote D’Ivoire, the prevalence of FGM was 15% amongst infants and young girls aged 1–14 years [17]. Roughly 87% of girls were documented to have Type II and 13% Type 1, according to the WHO classification for FGM. In a community-based study in Aysha, Kebribeyah, and Hartishek refugee camps in Eastern Ethiopia, the prevalence of FGM was 42% among Somali girls aged 1–12 years (n = 492) [18]. This rate was considerably higher amongst older girls, with 52% of girls 7–8 years and 98% experiencing FGM by 12 years of age [18]. 64% of girls were subjected to clitoral cutting and 36% vaginal stitching (infibulation). In a more recent study amongst purposely selected refugee adolescent girls (n = 260) in the Nakivale refugee settlement in Uganda, the prevalence of FGM was 10% [19]. Most of the circumcised girls were from Somalia (21 out of 27) with the average age of circumcision around 7 years of age [25]. The type of FGM was not recorded in this study. Two of these studies relied on self-reports of FGM, including type, while one relied on both self-report and physical examination.

Another three of the 17 studies synthesized programmatic data, internal assessments, and subnational studies on shifts in FGM practices in select locations [20–22]. Despite not collecting primary data, these publications were included in the review because they provided compelling data on shifts in FGM incidence in humanitarian settings from studies that were not readily available in the public domain. One study was conducted in South Sudan among Sudanese refugees who were displaced in 2011 and who had settled in refugee camps in Upper Nile State [21]. Those who came from Ad-Damazīn and Kurmuk in Sudan settled into what later became known as Doro Camp, while the ethnic Ingassana group settled into other camps including Batil and Gendrassa camps. Because FGM was a pre-existing practice in Sudan, there were strong suspicions among practitioners that FGM was occurring in the camps. The study thus examined FGM practices in Doro, Batil and Gendrassa camps and found that the practice appeared to be declining in Doro camp owing to awareness-raising campaigns that predated refugees’ arrival to South Sudan and due to shifts in social norms that no longer positioned FGM as a prerequisite to marriage. The practice ceased to be a social norm in Doro camp so much that it was no longer done openly out of fears of judgment from other community members. In contrast, in Batil and Gendrassa, prevalence did not appear to change because the social norms perpetuating the practice were found to be unaffected by displacement. In another assessment conducted in Uganda, evidence of a perceived increase in FGM incidence was generated through the use of a U-report poll, a mobile phone text messaging poll that collects data in real time from users. The poll, conducted in 2020, showed that 66% of respondents believed that FGM had increased because girls were increasingly staying at home due to the COVID-19 pandemic which had exacerbated economic pressures on parents [21]. There were additionally reports from Nigeria that FGM was increasing because former traditional excisors were resorting to providing FGM services to make money due to the limited economic opportunities available after the COVID-19 pandemic, as well as reported increases in the number of girls being cut across South-West Nigeria as a result of school closures during COVID-19.

### Factors associated with FGM in humanitarian settings

Ten articles addressed factors that contribute to FGM in humanitarian settings including a continued sense of social belonging and identity, concerns around marriageability, interaction with alternative perspectives, fears around legal restrictions against FGM in host countries, and medicalization of the practice.

A strong sense of social belonging and intergenerational influence of perpetuating the practice was described in a number of studies. The practice was often referenced as being ‘handed down’ through generations, often by mothers and female relatives [23–25]. This transfer was rooted in strong cultural and religious traditions [18, 23, 24], social pressure [24, 26], and the promotion of cleanliness [24, 27]. The desire to preserve social identity and group membership amplified in contexts of displacement, and in turn perpetuated the practice which is largely seen as an identity marker. As demonstrated by one study conducted with Somali refugees in Nairobi and Kenya, FGM was seen not only as a marker of religious identity but also national identity. Against a backdrop of systems and experiences of exclusion, “a strong connection with a personal gendered, religious, and national identity became a way of coping with marginalization” [26].

Frequently referenced, FGM was associated with marriage in displacement settings as a prerequisite, an attempt to preserve virginity and family honor, or control girls’ sexuality [17, 18, 23, 24, 26, 27]. Concerns around marriageability were particularly relevant in humanitarian contexts where there are limited opportunities for girls and where marriage is seen as the only option [23]. Fears around sexual violence and rape as well as concerns around sexual relations with foreign or non-Muslim men further positioned marriage as a way to preempt reputational damage and protect girls from the sexual violence rampant in humanitarian settings [26]. Indeed, in some cases, FGM Type III or infibulation was seen as a way to protect girls from rape [22]. Following marriage, FGM remained important for ensuring faithfulness on the part of the wife and her protection [27]. FGM and early forced marriage—which is common in humanitarian emergencies—were noted for their mutual reinforcement of one another [20].

Several studies revealed there was a transition from more harmfully perceived forms of FGM (pharaonic) to lesser forms (sunna) amongst the study participants [23, 26]. This transition was further described in one study as lessened by greater interaction with new and alternative perspectives on circumcision from a diverse community, including amongst medical providers unfamiliar with the practice [23]. In their study, Jinnah and Lowe find that “there was undoubtedly a move away from the extremity of pharaonic circumcision [FGM Type III]” among Somali women in Johannesburg, with women describing that they felt “free from social pressure” [26]. This was attributed to the reduced influence of intergenerational households in addition to greater interaction with non-Somalis. However, while interaction with new and alternative perspectives facilitated a transition from more severe to lesser forms of FGM in some cases, the opposite was observed in Mali where families displaced to the South of the country from the North where FGM is uncommon were adopting FGM due to pressures from the host communities and fears of being ostracized [20]. In this case, interaction with the host community had the opposite effect: it pushed families to adopt a new harmful social norm.

The medicalization of FGM through the widespread availability of health practitioners familiar with the practice in displaced communities [23, 26] was also discussed as an enabling factor. For example, in Nairobi, despite public health messages against FGM which drove Kenyan nurse-midwives to refrain from performing FGM or re-infibulating women, Somali nurses were “particularly sympathetic to women’s desires to be ‘closed’ again after delivery” and were thus more likely to conduct the procedure on Somali refugees living in the country. These traditional midwives often had little formal training and did not accept compensation [24].

Fears around the inability to perform FGM in host countries where there are strong legal restrictions were also found study to drive the practice. In one instance, practitioners found that Somali Bantu refugee parents in Kenya were circumcising girls as young as one and half before they were resettled to the United States out of fear of legal implications of FGM in the country of resettlement [22].

### Consequences of FGM in humanitarian settings

#### Health

A range of health consequences related to undergoing FGM surfaced across the ten articles that focused on consequences of FGM in humanitarian settings. Many of these consequences are not unique to humanitarian crises but because they occur against a backdrop of disrupted health and social services and limited access to emergency health care, they are more severe in magnitude and effect. Other consequences of FGM result from the increase in sexual violence and rape that occur in humanitarian crisis, and which have more dangerous implications for girls who are cut. Additionally, because health providers in host countries lacked knowledge of the risks and complications of FGM, these consequences were at times more pronounced.

The included studies identified immediate negative health outcomes related to fever [17]; infections, including of the reproductive and urinary tracts [24, 27, 28]; pain [17, 24]; minimal to excessive bleeding, depending on type of FGM [17, 24]; difficulty urinating [19, 24]; fistula; disfigurement of the vaginal area [24]; infectious diseases such as HIV/AIDS [24]; and death [27]. Also identified are various lifelong and ongoing consequences such as lower self-reported physical health [29]; menstrual pain [19]; painful sexual intercourse [24, 27]; low sexual desire [24]; diminished sexual pleasure [27]; and lack of pleasure or sensation during intercourse [24].

Serious health complications during childbirth were cited in several studies as FGM increased the risk of morbidity and mortality associated with pregnancy and delivery which were exacerbated in humanitarian settings in part due to providers’ lack of awareness of FGM and its impacts during delivery [28]. For birthing women who had undergone FGM, there is a higher risk of postpartum infections and complications during delivery including with dilation, scar tissue and the de-infibulation process [28]. In countries where FGM is uncommon, health providers are often unaware of the challenges confronting circumcised women during delivery and their higher risks for infections and complications. As observed by a Somali women interviewed in a study by Jinnah et al., “I tried to explain [to the nurses] what happens due to circumcision, but they don’t understand. They shout at us because we don’t push during childbirth, they don’t understand how hard it is for us to push. We can’t push.” [26].

Case studies recorded by “28 Too Many” further illustrate the negative consequences of FGM that amplify in crisis situations [20]. For example, in refugee camps in Sudan, girls who were raped and who were as young as ten were experiencing serious pregnancy-related complications because of their young age but also because they had undergone FGM, which further increased their already high risk for complications during childbirth [20]. As rape and sexual violence against girls occur at high rates in emergency situations, increases in childbirth complications associated with FGM are found to also increase [20].

#### Violence

Despite its correlation with other forms of violence, including domestic violence, sexual and physical abuse, polyvictimization, or the clustering of “interconnected risks of war-related, gender-based, and racially-motivated violence” was seldom used as a lens to understand the impacts of FGM and its relationship to other forms of violence [29]. An intersectionality lens that investigates the consequences of FGM for female refugees who embody several disadvantaged identities was only used in one of the ten articles. The study concluded that female refugees experience multiple and intersecting traumas emanating from various forms of violence which in turn increase their risk for developing lasting “mental, physical, and emotional problems” [29]. Indeed, Somali FGM survivors displayed in Kenya faced heightened risks—including risk of child and sexual abuse, compared to their counterparts who were not subjected to the practice. The intersection of different forms of violence was further evident in Nigeria where displaced women and girls were being cut to force them into prostitution [20].

#### Mental health and psychosocial wellbeing

Several mental health and psychosocial consequences related to FGM were cited across the studies and varied in type, clustering and severity of symptoms. Consequences ranged from less specific references to extreme emotional distress [24], heightened expression of symptoms related to depression, anxiety, post-traumatic stress disorder (PTSD), and somatoform pains [29]. The expression of each of these outcomes was differentially situated against the backdrop of multiple traumas that occurred throughout the migration journey, age at the time of FGM, and discussion about the practice before it occurred [29]. One study also revealed challenges for survivors related to socialization, suicidal ideation, and greater engagement with negative coping strategies including substance use [29].

### Interventions aiming to prevent FGM or to support survivors in humanitarian settings

Early attempts to respond to FGM in humanitarian action were described in relation to broader response to sexual and reproductive health needs of women in humanitarian crises using the Inter-Agency Field Manual (IAFM) for Reproductive Health in Refugee Situation [30, 31]. Developed through a global consortium of dedicated United Nations (UN) and non-governmental organizations (NGOs), the Inter-Agency Working Group on Reproductive Health in Crises (IAWG) released the Minimum Initial Service Package (MISP) for Sexual and Reproductive Health (SRH) as part of the 1999 IAFM [31]. Updated in 2010 and 2018, the MISP continues to be a lifesaving set of SRH services and activities that include guidance for service providers to support deinfibulation or referrals for services as needed for childbirth in humanitarian contexts. However, only these two aspects are noted explicitly in the MISP [32, 33].

Of the five studies identified, none were evaluations of the effectiveness of interventions aiming to prevent FGM or to support survivors in humanitarian settings. Most articles featured case studies on existing interventions or highlighted recommendations for developing or improving existing practice based on their analysis. Wenzel and colleagues use the lens of restorative and transitional justice to explore a pathway to ending FGM that emphasizes the use of community-based and traditional resolution models to holistically address the needs of survivors [34]. The use of participatory, localized, and/or bottom-up approaches were referenced by other studies that provide opportunities to integrate educational, health and other resources across a longer period of time [20, 21, 23]. The use of fully integrated services to support the survivor or girls at risk was commonly referenced as a key interventional approach [20, 24, 35]. Similarly, interventions must factor in supports not only for the girl but also across the social ecological system of support whereby including host and displaced populations in program design and decision-making [20, 29]. Involving men, religious and community leaders in programming was also highlighted [18].

Measurement was discussed as central to programme design and monitoring indicators on prevalence and consequences of FGM. One study suggested a situational analysis would be most helpful when jointly conducted by development and humanitarian actors. This would support the development of shared outcomes for long-term programming initiatives and tracking [21]. Another recommended that prevalence estimates and indicators on consequences should be collected using internationally standardized indicators to monitor trends and challenge misconceptions about the practice [24]. Enhanced monitoring of emergency response programming was also mentioned [20]. A case study revealed the potential to use digital innovation (U-Report) to identify cases or girls at risk in communities in real time in order to provide actionable information for local and national governments to respond [21]. Other digital solutions used in complex emergencies include tools for case management and incident monitoring, such as Primero and the GBVIMS+ [21].

Capacity development was described in relation to support directly for service provision and more generally to support humanitarian response. Interventions to support survivors included training to traditional birth attendants to identify complications in need of referral, and reduce likelihood of infections related to the use of handwashing and instrument sterilization for de-infibulation procedures [28]. Capacity development of those that respond to emergencies noted government actors, practitioners, and healthcare workers as critical in this pursuit [20].

Other interventions suggested throughout the literature relate to specific sectoral improvements. The strengthening of anti-FGM national laws was raised [18]. In some cases, this would require the establishment of a legal and policy framework for working with displaced populations and substantive political engagement [24]. Education and the dissemination of information, including on sexual and reproductive health, was highlighted by a few articles. Mainly, as it related to ensuring education of girls, families and communities [20, 24], and the importance of information required for active civil engagement and political debate to support transformation of social and cultural norms [24]. Another studies reflected on enhancing sanitation solutions for survivors to promote cleaner and more efficient waste disposal and proper hygiene [36].

## Discussion

Our scoping review set out to explore the landscape of research studies conducted on FGM in humanitarian settings and to understand dynamics around the practice in contexts where women and girls are particularly vulnerable. While there is a clear increase in the number of studies on FGM in humanitarian settings, most of which took place in the last decade, we found an overall dearth of literature investigating the impact of humanitarian emergencies on rates of FGM, and very few studies on the drivers and consequences of FGM in these settings. Moreover, none of the studies examined the effectiveness of interventions to prevent FGM or respond to the needs of survivors in contexts where community structures and services are disrupted. As a result, there are glaring gaps in the evidence base on how to address FGM in contexts of forced displacement and humanitarian crisis. Most studies that have investigated FGM among refugees and asylum seekers have done so in the context of migration to high-income countries, which fell outside the scope of this review, while very few studies examined the lived experiences of women and girls in humanitarian settings or among displaced populations residing in low- and middle-income countries. Why FGM has been understudied in humanitarian contexts is in part explained by the belief that more pressing priorities, such as food, shelter, and health, take precedence over perceived social issues which are believed to be more difficult to address in emergencies [21, 22].

While few studies investigated rates of FGM in humanitarian settings, those that did provide evidence that FGM is a deeply rooted practice perpetuated by social norms and cultural traditions that can transcend borders, as evidenced by the persistence of the practice after displacement. While evidence of the acquisition and transfer of FGM across populations is not conclusive, there is emerging evidence that families may adopt FGM due to social pressures and sanctions imposed by host community norms. Our finding that changes in the type of FGM from more radical to less extreme forms can take place is consistent with other literature that has documented shifts in FGM practices that occur after migrants settle in Western countries [37]. For example, Barrett et al. found that many migrants “stop performing FGM Type III but continue to perform FGM Types I, II and IV.” One explanation for this shift is the belief among migrants that milder forms like Type IV will be undetectable and thus unpunishable by law in high income countries where FGM is illegal [37]. In the context of low and middle-income countries, potential reasons for abandonment or shifts in FGM practices include diminished social pressure stemming from displacement into a new country where FGM is not a social norm, effective interventions against FGM, and the absence of excisors and medical personnel who are willing to conduct the procedure. Our findings merit additional research into how norms are transferred during migration flows and how social sanctions operate in humanitarian contexts to perpetuate or disrupt FGM.

The included studies suggest that the drivers of FGM may persist, intensify or attenuate in displacement contexts. For example, the studies demonstrate that there are drivers of FGM in humanitarian settings that may precede humanitarian crisis such as patriarchy and gender inequality—including reduced education and employment opportunities for girls, marriageability, aesthetics and lack of laws banning the practice. Other drivers are new or exacerbated due to crisis and displacement, such as a desire to preserve social identity, loss of livelihoods and economic insecurity, protection concerns, as well as disruptions in girls’ schooling and school closures. Crisis and displacement can also attenuate pre-existing drivers of FGM. The studies included in the review demonstrate that displacement may create a push and pull paradigm in the lives of girls. In some cases, displacement may reduce the influence of intergenerational households, thereby alleviating social pressures to cut girls. In other cases, it may enhance the desire for markers of social identity and increase risk of FGM. Moreover, displacement can disrupt the links between FGM and marriageability because social norms of the host community may not require girls to be cut. In other cases, where there are strong desires for social belonging compounded by elevated economic insecurity or protection concerns, girls may be cut to ensure early marriage is possible. Together, these findings highlight that the drivers of FGM in humanitarian settings are not uniform; instead, they are context-specific and may be affected by the social norms and practices of the host community.

The literature on consequences of FGM in humanitarian settings center around medical and health impacts that affect girls and women who are cut. A limitation noted in the overall literature on FGM including in non-humanitarian settings is a dearth of knowledge beyond medical consequences [5, 6]. Indeed, little is still known about how FGM, when initiated during critical and sensitive periods in a girl’s development, impacts the health and wellbeing over the life course. More research should be dedicated to exploring how adverse exposures in childhood—including FGM—can precipitate adaptive responses, such as an enhanced sense of social identity or belongingness, that may in the long run have deleterious effects on health, educational attainment, and employment opportunities. Furthermore, more should be done to examine the differential impact of transgenerational exposure to FGM, specifically in humanitarian settings, when structural approaches are well placed to shift social patterning of exposure, alleviate previous constraints prior to displacement, and provide opportunities for social and behavioral changes that can impact girls’ health positively.

Also missing in the literature is evidence on intervention effectiveness in humanitarian settings, with the majority of studies largely descriptive or theoretical. What the available literature indicates however is that community-based, integrated and multi-level intervention designs appear to be promising. Existing systematic reviews on the general effectiveness of FGM interventions are inconclusive [38, 39]. One review on FGM prevention in African countries found that the evidence base is too deficient to draw conclusions about that works to reduce the practice [39]. While successful in changing attitudes, none of the included studies in the review reduced the prevalence of FGM, which study authors attributed to poor implementation fidelity and weak study design. Similarly, a meta-analysis of studies to prevent FGM found limited effectiveness and an overall poor quality of available evidence [38]. Our review echoes conclusions from other studies that highlight the pressing need for research into how to effectively implement interventions and programs that prevent FGM and respond to the needs of survivors, particularly in humanitarian contexts where resources are overstretched, vulnerability is heightened, and attention is primarily directed to perceived life-saving interventions.

To our knowledge, this is the first scoping review conducted on FGM in humanitarian settings, and thus serves to summarize much needed data on the practice. We used a comprehensive search strategy and a systematic process for article selection and inclusion and conformed to standards for the conduct of scoping reviews. The findings however should be considered in light of limitations. For one, the exclusion of languages other than English, Arabic and French may have reduced the article pool. Since the majority of high-burden countries speak one of the three languages, we doubt that the language restriction impacted study selection. Second, we were constrained by the paucity of available evidence on the topic, particularly the lack of primary research, as well as poor and incomplete documentation. The studies included in the review focused on specific settings/refugee communities and so the evidence may not extend to other displaced or crisis-affected populations. Our review reveals important insights about the dynamics of FGM in humanitarian settings and demonstrates the need for more research and documentation of evidence to inform specific interventions and policies.

## Conclusions

Despite an increase in research on FGM in humanitarian settings, there is still a notable dearth of studies investigating the impact of emergencies on FGM and the social norms that propel it. Our review reveals important insights about the dynamics of FGM in humanitarian settings and demonstrates the need for more research and documentation of evidence to inform specific interventions and policies.

## Supplementary Information

**Additional file 1:** Search strategy for the scoping review.

## Data Availability

Data is available upon reasonable request from the corresponding author.

## Abbreviations

FGM: Female genital mutilation
PRISMA-ScR: Preferred Reporting Items for Systematic reviews and Meta-Analyses extension for Scoping Reviews
PTSD: Post-traumatic stress disorder
