# Supplementary material for: A scoping review of FGM in humanitarian settings: an overlooked phenomenon with lifelong consequences
Source: Confl Health. 2022 Sep 15;16:49. doi: 10.1186/s13031-022-00479-5 (PMC9476296; doi:10.1186/s13031-022-00479-5)
Supplement: Supplementary file 1 — Additional file 1: Search strategy for the scoping review. [file 13031_2022_479_MOESM1_ESM.docx]

**PUBMED**

“Circumcision, Female”[Mesh] OR “Female Circumcis*”[tw] OR “Infibulatio*”[tw] OR “Clitoridectom*”[tw] OR “Clitorectom*”[tw] OR “Female Genital Cut*”[tw] OR “Female Genital Mutil*”[tw]

AND

“refugees” [MESH] OR “refugee camps” [MESH] OR “ethnic violence” [MESH] OR “ethnic cleansing” [MESH] OR “relief work” [MESH] OR “disasters” [MESH] OR Conflict* [tw] OR “ethnic violence”[tw] OR genocide [tw] OR “relief work”[tw] OR disasters [tw] OR “refugee camp” [tw] OR “political violence” [tw] OR ethnocide [tw] OR “ethnic cleansing” [tw] OR “mass violence” [tw] OR “forced migration” [tw] OR “humanitarian cris*” [tw] OR “humanitarian setting*” [tw] OR “humanitarian emergenc*” [tw] OR “complex emergenc*” [tw] OR “Complex Humanitarian Emergenc*”[tw] OR “humanitarian response” [tw] OR “refugee setting*” [tw] OR displace* [tw] OR “humanitarian context*” [tw]

NOT

"case reports"[pt] OR "comment"[pt] OR "editorial"[pt] OR "legal case"[pt] OR "legislation"[pt] OR "newspaper article"[pt] OR "patient education handout"[pt] OR "retracted publication"[pt]

AND

"1990"[Date - Publication] : "3000"[Date - Publication]

**Embase**

(‘female genital mutilation’/exp OR ‘female circumcision’:ti,ab,kw OR ‘Female Circumcis*’:ti,ab,kw OR ‘Infibulatio*’:ti,ab,kw OR ‘Clitoridectom*’:ti,ab,kw OR ‘Clitorectom*’:ti,ab,kw OR ‘Female Genital Cut*’:ti,ab,kw OR ‘Female Genital Mutil*’:ti,ab,kw)

AND

('refugee'/exp OR 'refugee camp'/exp OR 'ethnic conflict'/exp OR 'ethnic cleansing'/exp OR 'relief work'/exp OR 'disaster'/exp OR 'conflict'/exp OR 'war'/exp OR 'genocide'/exp OR 'forced migration'/exp OR 'humanitarian crisis'/exp OR ‘Conflict*’:ti,ab,kw OR ‘ethnic violence’:ti,ab,kw OR genocide:ti,ab,kw OR ‘relief work’:ti,ab,kw OR disasters:ti,ab,kw OR ‘refugee camp’:ti,ab,kw OR ‘political violence’:ti,ab,kw OR ethnocide:ti,ab,kw OR ‘ethnic cleansing’:ti,ab,kw OR ‘mass violence’:ti,ab,kw OR ‘forced migration’:ti,ab,kw OR ‘humanitarian cris*’:ti,ab,kw OR ‘humanitarian setting*’:ti,ab,kw OR ‘humanitarian emergenc*’:ti,ab,kw OR ‘complex emergenc*’:ti,ab,kw OR ‘Complex Humanitarian Emergenc*’:ti,ab,kw OR ‘humanitarian response’:ti,ab,kw OR ‘refugee setting*’:ti,ab,kw OR ‘refugee camp*’:ti,ab,kw OR displace*:ti,ab,kw OR ‘humanitarian context*’:ti,ab,kw )

AND

([1990-2021]/py)

NOT

(Conference abstract: it OR letter: it OR editorial: it OR erratum: it OR Conference review: it OR Note: it OR Short survey: it OR Erratum: it)

**Web of Science**

((TS=(Infibulatio* OR Clitoridectom* OR Clitorectom* OR “Female Genital Cut*” OR “Female Genital Mutil*”)) AND TS=(Conflict* OR “ethnic violence” OR genocide OR “relief work” OR disasters OR “refugee camp” OR “political violence” OR ethnocide OR “ethnic cleansing” OR “mass violence” OR “forced migration” OR “humanitarian cris*” OR “humanitarian setting*” OR “humanitarian emergenc*” OR “complex emergenc*” OR “Complex Humanitarian Emergenc*”OR “humanitarian response” OR “refugee setting*” OR displace* OR “humanitarian context*” )) AND PY=(1990-2021)

**PsychInfo**

TX ( "Female Circumcis*” OR “Infibulatio*” OR “Clitoridectom*” OR “Clitorectom*” OR “Female Genital Cut*” OR “Female Genital Mutil*”" ) AND TX ( (MM "Refugees") OR (DE "War") OR (DE "Conflict") OR (DE "Disasters") OR “refugees” OR “refugee camps” OR “ethnic violence” OR “ethnic cleansing” OR “relief work” OR “disasters” OR Conflict* OR “ethnic violence” OR genocide OR “relief work” OR disasters OR “refugee camp” OR “political violence” OR ethnocide OR “ethnic cleansing” OR “mass violence” OR “forced migration” OR “humanitarian cris*” OR “humanitarian setting*” OR “humanitarian emergenc*” OR “complex emergenc*” OR “Complex Humanitarian Emergenc*” OR “humanitarian response” OR “refugee setting*” OR displace* OR “humanitarian context*” ) AND PY 1990-2021
